# Effectiveness of Flexible Ureterorenoscopy Versus Extracorporeal Shock Wave Lithotripsy for Renal Calculi of 5–15 mm: Results of a Randomized Controlled Trial

**DOI:** 10.1016/j.euros.2021.01.001

**Published:** 2021-02-02

**Authors:** Christian Daniel Fankhauser, Damian Weber, Michael Müntener, Cedric Poyet, Tullio Sulser, Thomas Hermanns

**Affiliations:** Department of Urology, University Hospital, University of Zurich, Zurich, Switzerland

**Keywords:** Renal stones, Urolithiasis, Ureterorenoscopy, Extracorporeal shock wave lithotripsy

## Abstract

**Background:**

Primary flexible ureterorenoscopy (URS) and extracorporeal shock wave lithotripsy (SWL) are treatment options in patients with renal calculi of 5–15 mm.

**Objective:**

To compare effectiveness, complication rates, and pain scores between primary URS and SWL.

**Design, setting, and participants:**

Between 2011 and 2016, patients with renal calculi between 5 and 15 mm were randomized to undergo either primary URS or SWL.

**Outcome measurements and statistical analysis:**

Stone-free rate and size of residual fragments assessed by computed tomography after 3 mo, complications, and pain scores were evaluated.

**Results and limitations:**

The study was prematurely closed after randomizing 44 patients due to poor accrual. The 3-mo stone-free rate and mean residual stone size were, respectively, 61% and 1.8 mm after URS and 48% and 2.4 mm after SWL. Early post-treatment pain scores were significantly higher after URS than after SWL on day 1 (3.3 vs 1.6, *p* =  0.02) and day 7 (5.2 vs 3.4, *p* =  0.04), but were no longer detectable after 3 wk and 3 mo, respectively. One Clavien-Dindo grade II complication was observed after URS (5%) and SWL (4%), while one (4%) grade IIIb complication was observed after SWL.

**Conclusions:**

URS appears to be associated with higher early post-treatment discomfort, which could be associated with routine postoperative stenting. Owing to premature closure of this trial, the power was insufficient to formally compare URS and SWL; however, the present data might be informative to counsel patients about treatment outcomes and allow future meta-analyses.

**Patient summary:**

This study was ended prematurely, but it contributes data about efficacy and side effects of different treatment options in patients with renal calculi.

## Take Home Message

Primary flexible ureterorenoscopy (URS) and extracorporeal shock wave lithotripsy (SWL) are both safe and effective treatment options in patients with renal calculi of 5–15 mm. Although this trial was closed prematurely and the power was insufficient to formally compare URS and SWL, the present data might be informative to counsel patients about treatment outcomes and allow future meta-analyses.

## 1 Introduction

Extracorporeal shock wave lithotripsy (SWL) and flexible ureterorenoscopy (URS) are the most common treatment options for kidney stones <15 mm. Following the introduction of SWL in the 1980s [1], it quickly became the gold standard for the treatment of kidney stones [2], [3]. In the 1990s, URS emerged as an alternative treatment option that had the advantages of direct visualization, disintegration, and extraction of kidney calculi [4], [5], [6]. Technical advances in URS in the last decade have resulted in a dramatic increase in the popularity of the treatment such that URS has become the new standard treatment in many centers. However, comparative evidence showing the superiority of URS over SWL is scarce.

## 2 Patients and methods

This randomized controlled single-center study was performed in a tertiary care academic center. Patients with newly diagnosed kidney stones appropriate for SWL and URS treatment, as assessed by abdominal computed tomography (CT), were invited to participate in the trial. The inclusion criteria were single or multiple kidney stones with a stone size >5 mm. The exclusion criteria were any stone size >15 mm, age <18 or >99 yr, pregnancy, current breast feeding, anticoagulation, ipsilateral ureterolithiasis, aneurysm of the aorta or renal artery, inability to position the patient on the SWL table (eg, due to severe skeletal deformity or morbid obesity), radiolucent stones that were not visible on ultrasound, or severe metabolic disturbances (eg, cystinuria, primary hyperparathyroidism, or renal tubular acidosis).

Patients eligible for study inclusion were informed about the trial; if they agreed to participate, they signed an informed consent form and were randomized into the SWL or URS arm. Balanced permutated block randomization occurred in blocks of six. The sequence of randomization was computer generated and was performed by the university hospital pharmacy using DatInf Randlist software v.1.2 (DatInf GmbH, Tübingen, Germany). Randomization data were kept strictly confidential in sealed envelopes that were accessible only to the primary and senior investigator. After randomization, the patient and the surgeon were informed about the intervention arm. The sample size calculation was performed based on an assumed clinically relevant difference in the stone-free rate of 20%. With an alpha of 5% and power of 85%, the sample size calculation yielded a necessary 107 patients per treatment arm. The study was approved by the local ethics committee (STV KEK-ZH 2011-0221/0) and registered on clinicaltrials.gov (NCT01514032). All included patients provided written informed consent, and the trial was conducted according to the provisions of the Declaration of Helsinki guidelines.

All patients were admitted to the hospital as inpatient cases for the treatments. The following preoperative parameters for each patient were recorded: age, gender, and stone size and location (upper calyx, middle calyx, lower calyx, pelvis, or multiple locations). Stone size was defined as the largest diameter of the largest stone, as measured on an abdominal CT scan.

SWL was performed using the Dornier DLS II lithotripter (Dornier MedTech, Wessling, Germany) without perioperative antibiotic prophylaxis. The exact stone location was identified by x-ray and/or ultrasonography, and was verified regularly during the treatment. In most cases, 3000 shock waves were applied to the kidney stone; fewer were applied if complete stone disintegration was observed earlier. A ureteric stent was not inserted.

Patients in the URS group received preoperative intravenous antibiotic single-dose prophylaxis with either trimethoprim-sulfamethoxazole (2 × 400/80 mg: 19 patients) or ciprofloxacin (400 mg; four patients). After cystoscopy and retrograde ureteropyelography, a guide wire was placed, and a 14 French ureteral access sheet (Flexor Parallel Rapid Release Ureteral Access Sheath; Cook Medical, Indianapolis, IN, USA) was inserted into the ureter. If primary access to the collecting system could not be achieved, a double J ureteric stent was placed for 14–21 d, and the patient was scheduled for a secondary URS. A flexible URS (7.5Fr and Flex-X; Karl Storz, Tuttlingen, Germany) was introduced. Irrigation was performed using a pressure-controlled combined irrigation/suction pump (Uromat E.A.S.I.; Karl Storz). Stone fragmentation was performed using a 30 W Sphinx holmium:YAG laser (LISA Laser Products, Katlenburg, Germany) if required. The laser settings were according to the surgeon’s preferences. All procedures were performed by a consultant or a resident as a teaching procedure. The stones or stone fragments were extracted using a stone basket. At the end of the procedure, all patients received a double-J ureteric stent, which was left in place for 7 d. The stent was removed on day 7 by cystoscopy or extraction string.

Treatment parameters, including operative time and intraoperative complications, were recorded for both groups. Patients underwent sonography of the kidneys and were usually discharged on postoperative day 1. Patients were seen in the outpatient clinic on day 7, as well as 3 and 12 wk after treatment. Pain was assessed by a numeric rating scale, ranging from 0 (no pain) to 10 (worst pain), on day 1 and at all follow-up visits. All perioperative complications up to 30 d after treatment were assessed using the Clavien-Dindo classification [7]. Stone-free status was determined by abdominal CT 3 mo after treatment.

The results for the continuous normally distributed variables were expressed as mean ± standard deviation, and differences in patient characteristics between the two groups were compared using Student unpaired *t* tests. Continuous non-normally distributed variables were presented as median and interquartile ranges, and were analyzed using a Mann-Whitney *U* test. The results for the categorical variables were presented as percentages, and the differences were analyzed using a Fisher’s exact test or a chi-square test where appropriate. A *p* value of <0.05 was considered significant. All statistical tests were two sided. Statistical analysis was conducted with IBM SPSS Statistics (version 21.0; IBM Corp., Armonk, NY, USA). The results were reported according to the Consolidated Standards of Reporting Trials (CONSORT) statement.

## 3 Results

In total, 44 of 165 (27%) eligible patients agreed to participate in the present study. Twenty-one patients (48%) were randomly selected to undergo SWL and 23 (52%) to undergo URS (Supplementary Fig. 1). After randomization of 44 patients over 4.5 yr, the study was terminated due to poor accrual. The baseline characteristics were well balanced between the two treatment groups (Table 1). Both treatment groups were comparable in terms of age (SWL: median 47 yr vs URS: 50 yr), gender distribution (24% vs 30%, female), and stone size (7.6 vs 8.1 mm). All patients were treated as allocated and followed as planned.

**Table 1:** Baseline characteristics of all patients randomized to URS and SWL treatment

	URS (*n* = 23)	SWL (*n* = 21)
Mean age (±SD)	50 (±13.2)	47 (±14.7)
Gender distribution, *n* (%)		
Female	7 (58)	5 (42)
Male	16 (50)	16 (50)
Median size of largest stone (mm)	8.1 (±2.4)	7.6 (±1.9)
Mean BMI (kg/m^2^)	27.2 (±4.6)	26.9 (±5.2)
Number of stones, *n* (%)		
1	15 (65)	16 (76)
2	7 (31)	3 (14)
3	1 (4)	2 (10)
Mean procedure time (±SD)	79 (±33)	50 (±8)

BMI = body mass index; SD = standard deviation; SWL = shock wave lithotripsy; URS = flexible ureterorenoscopy.

In patients treated by URS, successful primary ureteral access was achieved in 20 patients (87%), and the mean operating time was 79 ± 33 min. Three patients (13%) underwent URS after ureteric stent insertion. One URS procedure (4%) had to be discontinued due to impaired vision caused by bleeding but without the need for transfusion. Further, in four patients (17%), extravasation of the contrast medium was detectable on intraoperative fluoroscopy. All patients were treated successfully with prolonged stent placement.

In all patients treated by SWL, successful disintegration was reported after a median of 3000 (range 2500–4000) shock waves and a median procedure time of 50 ± 8 min. None of the patients who underwent SWL required intraoperative insertion of a ureteric stent.

Early post-treatment pain scores on day 1 were significantly higher after URS (3.3 ± 2.43 vs 1.6 ± 2.01, mean change score 95% confidence interval [CI] –3.24 to –0.322, *p* =  0.02). Pain scores on day 7 were significantly higher after URS than after SWL (5.2 vs 3.4, *p* =  0.04). No significant difference in pain scores between URS and SWL was detectable after 3 wk (1.5 vs 1.8, *p* =  0.754) and 12 wk (0.8 vs 0.5, *p* =  0.686), respectively. A return to work after 1 wk was observed in 11 of 15 patients (73%) after SWL and in nine of 18 patients (50%) after URS. A return to work after 3 wk was observed in 86% of patients after SWL and in 94% after URS.

The complications included one postoperative urinary tract infection (UTI; grade II complication) in each group (Table 2). Further, one patient (4%) was diagnosed with painful ureteral obstruction after passing fragments following SWL and subsequently underwent transurethral stent insertion (grade IIIb complication). Three months after surgery, 61% of patients in the URS and 48% in the SWL group were stone free (*p* =  0.55). The mean size of the stones in patients with residual stones was smaller after URS (1.83 vs 2.38 mm, *p* =  0.53).

**Table 2:** Primary and secondary outcomes

	URS (*n* = 23)	SWL (*n* = 21)	*p* value
Stone-free rate after 3 mo	14/23 (61%)	10/21 (48%)	0.55
Mean residual stone size (mm)	1.8	2.4	0.53
Pain scores			
Day 1	3.3	1.6	**0.02**
Day 7	5.2	3.4	**0.04**
Week 3	1.5	1.8	0.75
Week 12	0.8	0.5	0.67
Need for secondary URS	2 (8%)	–	
Complications			
Clavien grade II	1 (4%) (urinary tract infection)	1 (4%) (urinary tract infection)	
Clavien grade IIIb	0	1 (4%) (painful ureteral obstruction)	

SWL = shock wave lithotripsy; URS = flexible ureterorenoscopy.

Bold *p* values indicate statistical significance.

## 4 Discussion

In the present study, a nonsignificant trend for a higher stone-free rate in patients with kidney stones between 5 and 15 mm treated with URS, compared with SWL, was identified. Further, patients treated with URS were found to have smaller residual stones. Patients treated with URS had a lower 30-d complication rate, although early postoperative pain scores were significantly higher in the URS group; however, both groups reported low pain scores only.

To date, the efficacy of URS compared with SWL has been evaluated in a small number of randomized controlled trials (RCTs) and several cohort studies. Five recent RCTs and two recent meta-analyses point to the superiority of URS over SWL in patients with lower pole kidney stones [4], [5], [6], [8], [9], [10], [11]. However, research supporting this treatment for non–lower pole kidney stones is limited. To date, only two RCTs have included non–lower pole stones. The first study, similar to ours, failed to accrue the needed sample size and was terminated prematurely [12]. The second, which included obese patients only, found that URS showed a significantly higher stone-free rate than SWL (90% vs 68%) in a sample size of 46 patients [13]. The small number of patients and patient selection limit the generalizability of these studies. These published reports are in line with the results of the present randomized study, which suggests that URS has a higher stone-free rate in lower and non–lower pole stone studies, although data remain scarce for non–lower pole stones.

In addition, several retrospective cohort studies have suggested the superiority of URS over SWL. However, these studies are limited by their nonrandomized study designs [14], [15], [16], [17], [18], [19], [20], [21]. Further, most of these retrospective cohort studies were small, with a mean number of 162 patients, and statistical methods to control for confounders were not applied. Our group recently published the largest propensity score matched retrospective cohort study, with stone-free rates of 84% for URS and 71% for SWL [22]. These numbers are more optimistic than the results of the present randomized study. This discrepancy is most likely due to the stringent outcome assessment by CT employed in the present randomized study.

The most common complications following SWL and URS are UTIs. In the present cohort, 4% of all SWL and URS patients were diagnosed with a UTI, which is comparable with other SWL or URS series reporting UTI incidences between 0.5% and 2.5% [23], [24], [25] or between 6.4% and 7.7% [22], [26], [27], respectively. This highlights the importance of preoperative urine cultures, which should optimally be performed several days ahead of either procedure. While for SWL, European Association of Urology guidelines recommend prescribing perioperative antibiotics in patients with infected stones or bacteriuria only, it is recommended that perioperative antibiotics be given to every patient before URS [28]. However, choosing the right antibiotic and identifying the patients at risk of postoperative UTIs is challenging due to the poor correlation between cultures from voided urine and stone cultures [29].

The main complications of URS are ureteral injury or bleeding. In the present study, four (17%) minor ureteral injuries with extravasation of contrast medium were observed; however, all patients were managed successfully with prolonged stent insertion. This surprisingly high number could be due to the residents’ limited experience and the high proportion of patients undergoing URS without prestenting, as prestenting followed by secondary URS has been associated with a lower complication rate [30]. Although a recent study reported that URS can safely be performed in patients with anticoagulation [31], one procedure had to be aborted due to bleeding leading to impaired vision.

The complications of SWL in the present study included ureteral obstruction requiring stenting in one patient and an asymptomatic perirenal hematoma in another patient. Ureteral obstructions due to passing stones represent a known complication in around 4–8% of all patients undergoing SWL for kidney stones [22], [32], [33]. In our cohort, no patient developed symptomatic renal bleeding after SWL. This represents a dreaded complication but is reported only in <1% after SWL [34], [35].

Despite the limited sample size, a significant difference in postoperative pain in favor of SWL was observed in the present study. This could be caused by the procedure itself but also by the ureteral stenting, which is performed routinely after URS but not after SWL. Postoperative stenting has been shown to decrease postoperative reinterventions from 13% to 2% only in men undergoing URS for ureteral; however, the impact of stenting in men with kidney stones needs to be clarified in further studies [36]. A side effect of ureteral stents is postoperative morbidity, and the need for routine postoperative stenting is questioned by many, but a recent Cochrane Review highlighted the low level of evidence [37]. Given the results of this review, future randomized trials comparing SWL and URS should include an URS arm without postoperative stenting.

The major limitation of this trial is the limited sample size due to trial closure arising from poor accrual. Therefore, the insignificant results could be due to lack of power rather than missing differences. Trial closure due to insufficient accrual is a known problem in surgical trials, and the main barriers to recruitment are often patient and clinician perceptions and preferences [38].

## 5 Conclusions

Although the present study did not recruit the planned sample size, it adds evidence to the debate that URS could be a better treatment option in untreated kidney stones up to 15 mm in size. However, URS did not achieve a perfect stone-free rate, and the invasive nature of URS can lead to significant injury of the urogenital tract. Therefore, healthcare providers should carefully explain to patients that a certain percentage of patients who undergo URS may require more than one procedure. Similarly, patients should be counseled on the limited stone-free rate of SWL and the potential of ureteral obstruction due to passing stones, leading to flank pain and the need for stent insertion. Further, perirenal hematoma can occur, although most cases remain asymptomatic. Symptomatic hematoma or prolonged bleeding represents a rare occurrence following SWL. Although definite final conclusions can be drawn, we believe that the outcomes of patients enrolled in this trial could be used for future meta-analyses.

  ***Author contributions*:** Christian Daniel Fankhauser had full access to all the data in the study and takes responsibility for the integrity of the data and the accuracy of the data analysis.

  *Study concept and design*: Müntener, Weber, Hermanns.

*Acquisition of data*: Weber, Fankhauser.

*Analysis and interpretation of data*: Fankhauser, Hermanns, Müntener, Weber.

*Drafting of the manuscript:* Fankhauser, Hermanns.

*Critical revision of the manuscript for important intellectual content:* Sulser, Poyet.

*Statistical analysis:* Fankhauser.

*Obtaining funding:* None.

*Administrative, technical, or material support:* None.

*Supervision:* AP, VS.

*Other*: None.

  ***Financial disclosures*:** Christian Daniel Fankhauser certifies that all conflicts of interest, including specific financial interests and relationships and affiliations relevant to the subject matter or materials discussed in the manuscript (eg, employment/affiliation, grants or funding, consultancies, honoraria, stock ownership or options, expert testimony, royalties, or patents filed, received, or pending), are the following: None.

  ***Funding/Support and role of the sponsor*:** None.

  ***Ethics statement*:** The study was approved by the local ethics committee (STV KEK-ZH 2011-0221/0) and registered on clinicaltrials.gov (NCT01514032).

  ***Data sharing*:** Data are provided on reasonable request.

## CRediT authorship contribution statement

**Christian Daniel Fankhauser:** Formal analysis, Data curation, Writing - original draft. **Damian Weber:** Methodology, Validation, Investigation, Data curation, Writing - review & editing, Project administration. **Michael Müntener:** Conceptualization, Methodology, Validation, Investigation, Data curation, Writing - review & editing, Project administration. **Cedric Poyet:** Validation, Investigation, Writing - review & editing. **Tullio Sulser:** Validation, Investigation, Resources, Writing - review & editing, Supervision. **Thomas Hermanns:** Writing - original draft.
