# Supplementary material for: Effectiveness of Flexible Ureterorenoscopy Versus Extracorporeal Shock Wave Lithotripsy for Renal Calculi of 5–15 mm: Results of a Randomized Controlled Trial
Source: Eur Urol Open Sci. 2021 Feb 2;25:5–10. doi: 10.1016/j.euros.2021.01.001 (PMC8317856; doi:10.1016/j.euros.2021.01.001)
Supplement: Supplementary file 1 [file mmc1.docx]

**Supplementary Figure 1** – CONSORT diagram of patient flow. SWL: shock wave lithotripsy, URS: flexible ureterorenoscopy
