# Supplementary material for: Effectiveness of Flexible Ureterorenoscopy Versus Extracorporeal Shock Wave Lithotripsy for Renal Calculi of 5–15 mm: Results of a Randomized Controlled Trial
Source: Eur Urol Open Sci. 2021 Feb 2;25:5–10. doi: 10.1016/j.euros.2021.01.001 (PMC8317856; doi:10.1016/j.euros.2021.01.001)
Supplement: Supplementary file 2 [file mmc2.pdf]

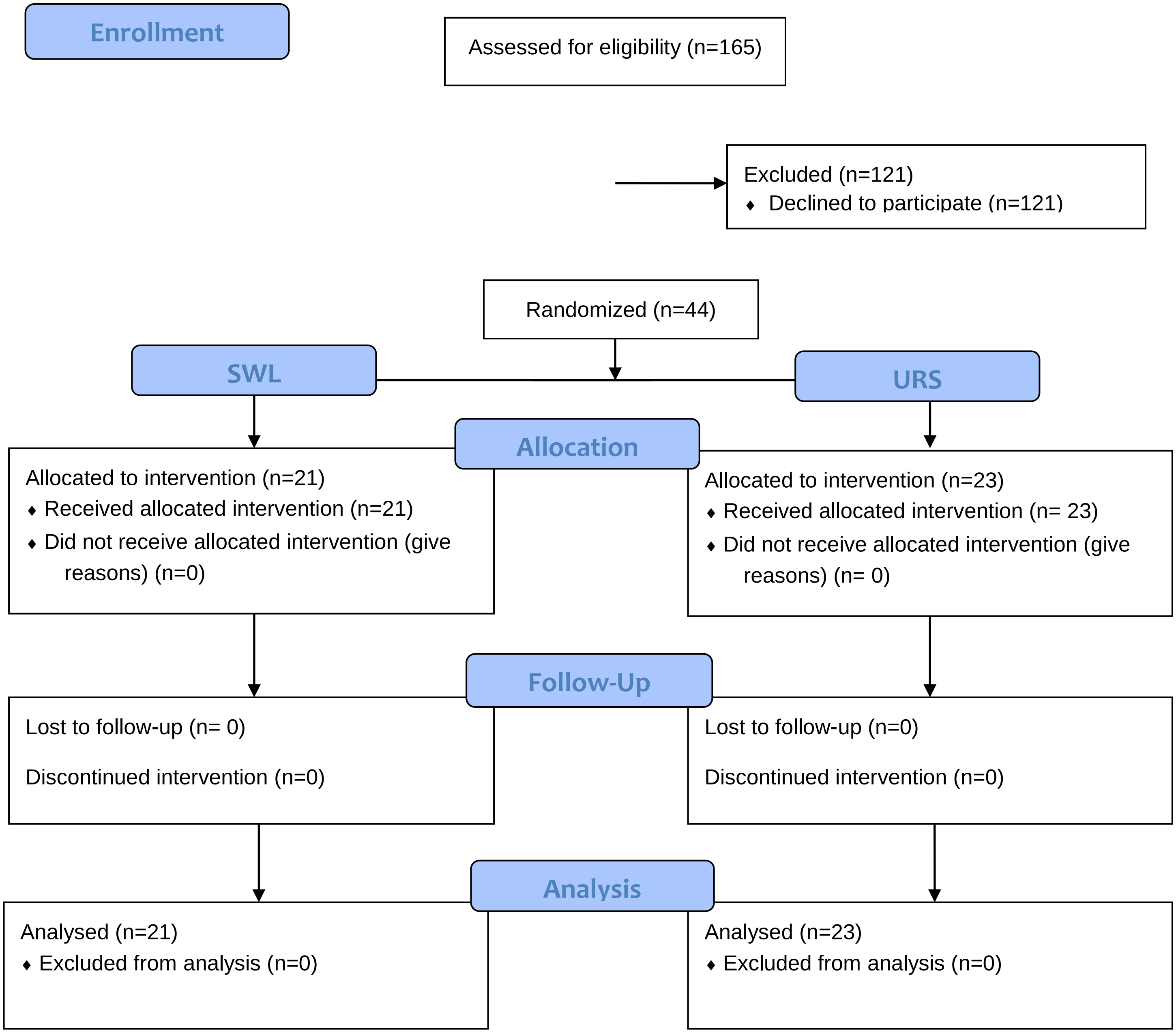
Enrollment
Assessed for eligibility (n=165)
Excluded (n=121)
Declined to participate (n=121)
Randomized (n=44)
SWL
URS
Allocation
Allocated to intervention (n=21)
Received allocated intervention (n=21)
Did not receive allocated intervention (give reasons) (n=0)
Allocated to intervention (n=23)
Received allocated intervention (n= 23)
Did not receive allocated intervention (give reasons) (n= 0)
Follow-Up
Lost to follow-up (n= 0)
Discontinued intervention (n=0)
Lost to follow-up (n=0)
Discontinued intervention (n=0)
Analysis
Analysed (n=21)
Excluded from analysis (n=0)
Analysed (n=23)
Excluded from analysis (n=0)
